# Exploring bioactive phytochemicals as ULK1 activators for enhancing cytoprotective autophagy in amyotrophic lateral sclerosis

**DOI:** 10.3389/fphar.2025.1661744

**Published:** 2025-09-04

**Authors:** Farah Anjum, Nahed Hawsawi, Abdulraheem Ali Almalki, Anas Shamsi, Maram Jameel Hulbah, Maha Bakhuraysah, Abdulaziz Alsharif, Taj Mohammad

**Affiliations:** ^**1**^ Department of Clinical Laboratory Sciences, College of Applied Medical Sciences, Taif University, Taif, Saudi Arabia; ^2^ King Salman Center for Disability Research, Riyadh, Saudi Arabia; ^3^ Centre of Medical and Bio-Allied Health Sciences Research, Ajman University, Ajman, United Arab Emirates; ^4^ Centre for Interdisciplinary Research in Basic Sciences, Jamia Millia Islamia, New Delhi, India

**Keywords:** amyotrophic lateral sclerosis, autophagy, UNC-51-like kinase 1, small molecule activators, virtual screening, candidine, delavinone

## Abstract

**Background:**

Amyotrophic lateral sclerosis (ALS) is a fatal neurodegenerative disorder that results in the degeneration of motor neurons and is typically linked to toxic aggregates of mutant superoxide dismutase 1 (SOD1) protein. As autophagy is critical for the removal of these toxic protein aggregates, stimulating autophagy has emerged as a promising therapeutic approach for ALS. Unc-51-like kinase 1 (ULK1) is a key regulator of autophagy and has been shown to have the potential to prevent ALS pathology when activated. However, synthetic ULK1 activators are frequently limited by toxicity and suboptimal pharmacokinetic profiles. This study aimed to identify natural ULK1 activators using a systematic virtual screening approach for potential ALS therapy.

**Materials and Methods:**

This study employed a comprehensive virtual screening approach to identify phytochemicals capable of activating ULK1. Natural compounds from the IMPPAT database were screened using molecular docking, followed by pan-assay interference compounds (PAINS) filtering, pharmacokinetic profiling, and density functional theory (DFT) analysis. Further, biological activity was predicted using the PASS tool, and candidate molecules were subjected to molecular dynamics (MD) simulations, essential dynamics, and binding free energy calculations via MM-PBSA.

**Results:**

The systematic screening in this study identified Candidine and Delavinone as high-affinity binders with reference to BL-918, proposing them as potential activators of ULK1. Both compounds demonstrated favorable drug-likeness, stable interactions with ULK1 in MD simulations, and promising ALS-relevant activity profiles. Essential dynamics and MM-PBSA further supported the binding stability and energetic favorability of these interactions.

**Conclusion:**

Candidine and Delavinone emerge as promising phytochemical activators of ULK1 with potential therapeutic relevance for ALS. These findings warrant further experimental validation and preclinical studies to explore their efficacy in autophagy modulation and neuroprotection.

## 1 Introduction

Amyotrophic lateral sclerosis (ALS) is a fatal, progressive degenerative disorder of the motor system, affecting primarily motor neurons within the brain and spinal cord, resulting in weakness in the muscles, paralysis, and eventual respiratory failure (Feldman et al., 2022). It is a complex disease characterized by the selective degeneration of upper and lower motor neurons, resulting in the loss of voluntary muscle control and severe functional impairments (Nijssen et al., 2017). The incidence rate of ALS worldwide is about 1–2 per 100,000 people, and most patients with ALS die within 2–5 years after the onset of the disease (Xu et al., 2020). There are sporadic (sALS) and familial (fALS) forms of the condition, and 10% of cases are familial (Hewitt et al., 2010). The insight into the pathology of fALS provided by genetic mutations associated with the disease has identified *C9orf72*, *SOD1*, *TARDBP*, and *FUS* as the most prevalent known contributing genes (Jankovska and Matej, 2021). Yet, while the genetic and molecular pathways responsible for ALS have been defined, the mechanisms by which ALS progresses remain poorly understood, and until now, there is no cure (Ruffo et al., 2025).

Misfolded and aggregated proteins in motor neurons are a defining pathological feature of ALS (Duranti and Villa, 2022). Among these, mutant superoxide dismutase 1 (SOD1) aggregates are extensively studied and are known to play a significant role in neuronal toxicity (Kaur et al., 2016). As a result, the expected proteasomal degradation of misfolded SOD1 proteins typically forms insoluble aggregates that activate endoplasmic reticulum (ER) stress, mitochondrial dysfunction, and oxidative damage, ultimately leading to motor neuron degeneration (Chen et al., 2021). Moreover, these deleterious aggregates also impede critical cellular functions, such as RNA metabolism, axonal transport, and synaptic transmission. Accumulation of misfolded proteins is a hallmark of ALS pathology; thus, targeting the clearance of these misfolded and potentially toxic protein species is an attractive therapeutic avenue (Duranti and Villa, 2022).

Autophagy is a highly conserved intracellular pathway that cleaves and recycles damaged proteins and organelles, thereby contributing to the maintenance of cellular homeostasis (Chun and Kim, 2018). It is characterized by the sequestration of cytoplasmic cargo into double-membrane, cytosolic structures called autophagosomes, which then fuse with lysosomes for degradation. Autophagic flux is dysregulated in ALS patients, and multiple preclinical models and a loss of autophagic clearance of toxic protein aggregates have been documented (Ramesh and Pandey, 2017). This inappropriate autophagic impairment results in further accumulation of misfolded proteins and a vicious cycle of cellular stress-mediated neurodegeneration (Esmaeili et al., 2022). This has led to considerable interest in therapeutic strategies that activate autophagy, particularly as a treatment for ALS (Chua et al., 2022).

Unc-51-like kinase 1 (ULK1) is a serine/threonine kinase and a key initiating enzyme in autophagy (Zou et al., 2022). The integration of upstream energy and nutrient signaling is mediated by the ULK1 complex, which comprises ULK1, ATG13, FIP200, and ATG101 (Wong et al., 2013). Under cellular stress, ULK1 is activated, leading to the formation of autophagosomes that clear damaged proteins and organelles (Zachari and Ganley, 2017). Importantly, ULK1 is a critical regulator of autophagy induction; therefore, modulating its activity may lead to an effective improvement of autophagic flux in ALS (Liu et al., 2023). Several studies have demonstrated that *C9orf72*, the most commonly mutated gene in ALS, interacts with the ULK1 complex and mediates autophagy (Yang et al., 2016). A mutation in C9orf72 causes loss of function, resulting in defective autophagic signaling, protein aggregate accumulation, and increased neuronal vulnerability (Chua et al., 2022). Increased ULK1 expression, along with its phosphorylation by AMPK at Ser317 and Ser777, triggers the activation of autophagy. Consequently, pharmacological and genetic activation of ULK1 stimulates autophagy and reduces neurotoxicity in ALS models, providing a strong rationale for the development of ULK1-targeted therapies (Vahsen and Lingor, 2021; Liu et al., 2023). Currently, no FDA-approved ULK1-targeted drugs exist for ALS, reinforcing the urgency for identifying novel candidates like BL-918 and phytochemicals in this study.

While some synthetic ULK1 activators have been investigated, their clinical utility is typically limited by toxicity, undesired pharmacokinetics, and low therapeutic potency (Zhang et al., 2017b; Ouyang et al., 2018; Liu et al., 2023). Thus, for the activation of ULK1 with minimal side effects, there is a need for novel, safer, and more efficacious compounds. Considering the structural diversity and bioactive properties of natural compounds, especially phytochemicals, they are potential alternatives (Ali et al., 2024). Natural products have been identified as a promising source of bioactive compounds with therapeutic properties for targeting complex diseases, including ALS, neurodegeneration, and cancer (Mohammad et al., 2020; Rahman et al., 2021). The phytoconstituents of medicinal plants are reported to have a wide range of chemical structures with various biological actions (Saxena et al., 2013). Thus, they can be an important source of leads for new drugs. Since natural compounds have been documented as modulators of autophagy, some of them are able to confer neuroprotective effects, mediated by both reducing oxidative stress and inflammation as well as facilitating protein clearance (Chandrasekaran et al., 2023). Recent recognition of ULK1 as a target for ALS therapy has led to the development of new strategies that enable a systematic screening for phytochemicals with the potential to boost ULK1 activity in a manner that could translate into an essential therapeutic agent for ALS.

Computational drug discovery methods have significantly accelerated the identification of new drug candidates (Naithani and Guleria, 2024; Tripathi et al., 2024). Methods such as virtual screening and molecular docking enable the rapid identification of small molecules that exhibit high-affinity binding to specific protein targets, significantly reducing the time and costs associated with traditional drug screening (Khan et al., 2022). Virtual screening is a systematic process used to screen large compound libraries, filtering for candidates that may structurally resemble the target protein and possess favorable predicted pharmacokinetic properties (Xue et al., 2022). These studies are conducted through further experimentation and typically include toxicological studies, such as absorption, distribution, metabolism, excretion, and toxicity (ADMET) studies, to assess the drug-likeness and toxicity of a compound (Shukla et al., 2018). Screening involves various computational approaches, including molecular docking and molecular dynamics (MD) simulations, to filter candidates and determine details about the binding stability and poses of the drug candidate within the binding pocket of the target protein (Shukla and Tripathi, 2020; 2021).

Using a multi-tiered virtual screening approach, this study selected candidate bioactive phytoconstituents relevant for initiating biological activities that could potentially induce ULK1 activation. A diversity of phytochemicals was extracted from the IMPPAT 2.0 database (Vivek-Ananth et al., 2023) and filtered for drug-likeness parameters using the Lipinski rule of five (Lipinski, 2000). Initial molecular docking studies were performed to screen compounds with potentially high binding affinity to ULK1. Subsequently, ADMET analysis, as well as the PAINS filter [33], were employed to identify suitable pharmacokinetic and toxicological profiles. Selected compounds were further investigated for potential biological activities using the Prediction of Activity Spectra for Substances (PASS) tool (Khan et al., 2022). The subsequent filtration of these top-scoring candidates involves subjecting them to all-atom MD simulations, followed by essential dynamics analysis to assess their binding stability and interaction time frame with ULK1. Collectively, this identification of natural compounds that stimulate ULK1-mediated autophagy may provide a basis for developing targeted molecular mechanisms to inhibit neurodegeneration in the context of ALS. Since ALS is a disease for which there is an unmet clinical need for effective therapies, bioactive phytoconstituents targeting ULK1 present a promising avenue for future drug development against this devastating disease.

## 2 Materials and methods

### 2.1 Computational tools and web resources

Several bioinformatics tools and online resources were used in this virtual screening approach to identify potential activators of ULK1. Molecular docking was performed to predict binding affinities between phytoconstituents and ULK1 protein using InstaDock v1.2 (Mohammad et al., 2021). The docking screening generated output evaluating the interaction strength and orientation of each compound within the ULK1 binding site. Complex protein-ligand complexes were generated for structural visualization and interaction analysis using PyMOL (DeLano, 2002) and Discovery Studio Visualizer (Visualizer, 2005) in a three-dimensional (3D) and two-dimensional (2D) format. Deep-PK (Myung et al., 2024) was used to predict the pharmacokinetic parameters of the screened compounds, assessing their ADMET properties. The PASS server (Lagunin et al., 2000) was used to explore the potential biological activities of the compounds. The phytochemical dataset was retrieved from the IMPPAT 2.0 (Indian Medicinal Plants, Phytochemistry, and Therapeutics Database), a database that contains plant-derived bioactive compounds (Vivek-Ananth et al., 2023). The IMPPAT 2.0 database was chosen due to its comprehensiveness and curation, featuring over 18,000 phytochemicals from Indian medicinal plants. It includes 3D structures, ethnopharmacological relevance, and drug-likeness annotations, making it highly suitable for virtual screening in neurological disorders like ALS.

### 2.2 Receptor and library preparation

The RCSB Protein Data Bank (Burley et al., 2022) was used to retrieve the protein structure with PDB ID 6QAS, having a resolution of 1.75 Å (Chaikuad et al., 2019). Co-crystallized ligands and water molecules were removed from the structure using PyMOL software, and the structure was refined to prepare it for molecular docking. Prior to docking, the ULK1 protein structure was energy minimized using Swiss-PDB Viewer to resolve steric clashes and ensure structural optimization. PDB ID 6QAS was selected for its structural completeness, co-crystallized ligand reference, and optimal resolution of 1.75 Å without mutation. Other structures like 4WNO (1.56 Å) were avoided due to introduced mutations that could bias docking/simulation interpretations. The phytochemical library was extracted from the IMPPAT 2.0 database, which contains 3D conformations in PDBQT file format. Compliant with Lipinski’s Rule of Five for drug-likeness and bioavailability, compounds were filtered based on molecular weight, lipophilicity, and the stereochemistry of hydrogen bonds. Molecules were energy-minimized to avoid steric clashes and optimize structural conformation using the Swiss-PDB Viewer (Kaplan and Littlejohn, 2001). The processed compounds were then uploaded into InstaDock for molecular docking screening against ULK1. Ligand Efficiency (LE) was calculated as the docking score divided by the number of heavy (non-hydrogen) atoms.

### 2.3 Molecular docking screening

Molecular docking is a widely used computational technique for identifying compounds that interact with the binding site of a target protein (Hassan et al., 2017). It predicts the binding affinity and ligand efficiency of molecules for use as potential drug candidates. Structure-based virtual screening expedites this process by rapidly analyzing extensive compound libraries to identify suitable candidates based on their docking scores and interaction features with a specific target. InstaDock v1.2 was used for docking screening in a defined search space to identify compounds with potential binding sites on the ULK1 protein (Mohammad et al., 2021). The grid was defined with an X dimension of 28 Å, a Y dimension of 32 Å, and a Z dimension of 22 Å based on the predefined binding site (Liu et al., 2023). The center was positioned at coordinates 7.88 −3.917 and 21.722 to encompass the entire protein surface. A grid spacing of 1 Å was used to ensure precise coverage. The receptor was used as a three-dimensional structure of ULK1, and compounds from the phytochemical library were docked to determine their affinities. The output files were extracted from the docking results, and the compounds were compared based on their binding scores. The best candidates with promising binding affinities were then identified for subsequent evaluations. To validate the docking protocol, the co-crystallized ligand in 6QAS was re-docked into the binding site using InstaDock. The RMSD between the docked pose and the crystal structure was 0.897 Å, indicating good agreement and validating the docking protocol.

### 2.4 ADMET and PAINS prediction

In drug discovery, the pharmacokinetic and toxicity profiles of compounds are crucial in determining their suitability for therapeutic use. Here, Deep-PK (Myung et al., 2024) was used to characterize the ADMET properties of the screened compounds from the docking analysis. Deep-PK thresholds aligned with CNS drug development requirements, focusing on high GI absorption, BBB permeability, and CYP450 inhibition avoidance. The carcinogenic potential of these compounds was evaluated using the CarcinoPred-EL web server (Zhang et al., 2017a). PAINS filtering was applied to avoid Pan-assay interference compounds and reduce the number of false positives, thereby selecting compounds with better pharmacokinetic properties and reduced toxicity risks.

### 2.5 PASS analysis

PASS (https://www.way2drug.com/passonline/) is a web-based application that predicts the biological activity spectrum of a compound based on its molecular structure (Lagunin et al., 2000). PASS analysis was performed to explore the potential biological activities of the screened compounds. PASS analysis predicts the active (Pa) or inactive (Pi) probability of a compound for a particular biological function. This suggests that a higher Pa value demonstrates an increasing likelihood of biological activity. PASS analysis was used to determine if there is a possible correlation between the shortlisted compounds and ULK1 activation in ALS, allowing for the prioritization of promising candidates for further assessment. For PASS, predicted activities were accepted if Pa > Pi, indicating functional relevance.

### 2.6 Interaction analysis

After PASS analysis, molecules with promising results were then subjected to molecular interaction studies to determine their binding to the ULK1 binding pocket. Ligand-protein interactions were visualized from docking output files obtained using InstaDock. Molecular interactions were examined using PyMOL (DeLano, 2002) for the screened molecules, and 3D and 2D diagrams were drafted in Discovery Studio Visualizer (Visualizer, 2005) to assess various interactions, including hydrogen-bonding, hydrophobic interactions, and π-π stacking. The interactions that occurred within the ULK1 binding site between the compounds were the most favorable. To get more detailed information about the stability and dynamic behavior of the selected compounds in the ULK1 complex, we performed MD simulations so that we could explore the binding orientation and structural stabilities of the ligand-protein at the timescale of the simulation. BL-918 was selected as a reference molecule due to its validated ULK1 activation activity and reported efficacy in ALS models, serving as a pharmacological benchmark. The top binding pose was selected based on the lowest docking score, favorable hydrogen bonding, and structural similarity to BL-918 within the ULK1 binding site.

### 2.7 Density functional theory

To evaluate the electronic characteristics of the selected compounds, density functional theory (DFT) calculations were conducted using the ORCA software suite (version 6.0.1) (Neese et al., 2020). Molecular structures were constructed in the Avogadro 1.2 package (Hanwell et al., 2012), and input files for calculations by DFT were prepared in XYZ format. Geometry optimizations were carried out using the B3LYP hybrid functional (Becke, 3-parameter, Lee-Yang-Parr) with tight convergence for SCF cycles. Vibrational frequency calculations were performed for each optimized geometry to ensure that each structure represented a true energy minimum and that the thermodynamic parameters (energy and entropy) were obtained. From this study, important electronic descriptors including the highest occupied molecular orbital (HOMO), lowest unoccupied molecular orbital (LUMO) and optimized geometries were obtained.

### 2.8 MD simulations

MD simulation is a method for analyzing the time-dependent (dynamical) behavior of atoms and molecules in the context of Newtonian mechanics (Shukla and Tripathi, 2020). Understanding protein-small molecule interactions, including complex stability and dynamic conformational changes, is crucial for drug discovery; therefore, MD simulations are utilized to gain deeper insights. MD simulations of ULK1 and its complexes with selected compounds were conducted using the GROMACS 2020 β suite (Van Der Spoel et al., 2005). The GROMOS 54A7 force field (Schmid et al., 2011) was used in this MD simulation to define atomic interactions, allowing for accurate energy calculations and MD predictions. Individual molecular structures were processed for MD simulation using the PRODRG web server (Zhu, 2019) to obtain force field parameters for the screened compounds. A water model SPC216 (Mark and Nilsson, 2001) was used to solvate the systems. Each system was confirmed to be well-structured through energy minimization using the steepest descent method, which avoided steric clashes and enabled the achievement of a stable starting structure. The system was then equilibrated by increasing the temperature from 0 K to 300 K over a period of 100 ps to stabilize molecular motion following energy minimization. Equilibration was performed in two steps using the NVT (number of particles, volume, temperature) ensemble to simulate 100 ps, ensuring temperature equilibration, followed by the NPT (number of particles, pressure, temperature) ensemble, which is similar to NVT but allows the system’s pressure and density to change. This ensured that the system remained thermodynamically stable under the conditions of the production run. Each system was then run to study the time evolution of the molecules for at 300 ns. Various parameters, including solubility, binding free energy, kinetic stability, and structural integrity of ULK1-ligand complexes, were evaluated. While these observations define the molecular engagement of candidate compounds with ULK1, they provide additional evidence supporting their assessment as potential drug candidates.

### 2.9 Principal component analysis

Principal component analysis (PCA) is a widely used statistical technique in computational biology, as well as chemistry and structural bioinformatics (Yang et al., 2009). It is typically applied to remove high-dimensional complexity in datasets while retaining the most relevant information about the variations of a system. When it comes to results from MD simulations, PCA is applied to summarize atomic fluctuations from MD simulations and extract a few collective motions in the large-scale conformational changes of biomolecules. This method can then determine the dominant modes of motion that inform protein flexibility and rigidity before and after ligand complex formation. PCA was performed on the MD trajectories using *the gmx covar* and *gmx anaeig* modules of GROMACS software to investigate the essential dynamics of ULK1 in both unbound and bound states with the selected phytoconstituents. The first few principal components can describe most of the atomic fluctuations, which can be considered to characterize the dynamics and stability of the protein-ligand molecular complex.

### 2.10 Free energy landscapes

Thermodynamic stability and folding dynamics of ULK1 and its complexes were analyzed using free energy landscape (FEL) analysis (Abdelsattar et al., 2021) with the *gmx sham* module in GROMACS. We subsequently employed the FELs to assign the conformational states of low-Gibbs-energy systems as a function of the projections of their principal components. Our studies reveal the stable and metastable states of ULK1 and how ligand binding reshapes the global energy landscape representation of ULK1. We next evaluated the stability of scaffolding, conformational transitions, and ligand-induced allosteric modulation of free versus ligand-bound ULK1 by analyzing the FELs of both states. FEL analysis of PCA integrated within FEL analysis provided significant insights into ULK1 activation, particularly about physiological motions and energy landscapes of ULK1.

### 2.11 MM-PBSA analysis

The binding free energies of protein-ligand complexes were evaluated by the Molecular Mechanics Poisson–Boltzmann Surface Area (MM/PBSA) method (Genheden and Ryde, 2015). We used the gmx_MMPBSA program to carry out four of these calculations over selected trajectory fragments that stemmed from GROMACS simulations (Valdés-Tresanco et al., 2021). To do so, we extracted 10-ns segments from each protein-ligand complex trajectory of a 300-ns production run and performed energy evaluations every 0.1 ns. This via MM/PBSA approach allowed for decomposing the various energy components such as van der Waals terms, electrostatic terms, internal molecular energies, the polar and nonpolar solvation, and the electrostatic component of the solvation. Δ*G*_binding_ is calculated from the equation:

$$∆G_{binding}=G_{complex}\;–\;\left(G_{receptor}+G_{ligand}\right)$$

where *G*_complex_ is the total energy of the protein-ligand system, *G*_receptor_ is the energy of the isolated protein, and *G*_ligand_ represents the energy of the unbound ligand.

## 3 Results

### 3.1 Molecular docking screening

A systematic virtual screening was conducted on a phytochemical dataset comprising ∼18,000 compounds derived from the IMPPAT 2.0 database. The first stage of this screening process consisted of applying the Lipinski rule of five to filter the compounds, which defines drug-likeness in terms of molecular weight, hydrogen bond donors and acceptors, and lipophilicity. This screening resulted in a filtered library of 11,908 phytochemicals. These phytochemicals were docked with the ULK1 protein using InstaDock to determine correlated binding affinities. Docking analysis yielded different conformations of each ligand, ranked by their binding energies and interactions within the ULK1 binding site. The compounds were ranked based on docking scores, and the top 10 compounds were selected, with binding affinities ranging from −11.0 to −10.3 kcal/mol, indicating a promising interaction with ULK1 (Table 1). Importantly, all chosen compounds exhibited considerably stronger binding affinities compared to the reference ULK1 activator, BL-918 (Liu et al., 2023), which has an affinity of −9.4 kcal/mol.

**TABLE 1:** Top 10 phytoconstituents and their docking scores against ULK1.

S. No.	Phytochemical ID	Phytochemical name	Source (plant)	Affinity (kcal/mol)	Ligand efficiency
1	IMPHY009120	Withametelin B	*Datura metel*	−11.0	0.3333
2	IMPHY006882	Candidine	*Strobilanthes cusia*	−10.6	0.3786
3	IMPHY007679	Bismurrayaquinone A	*Murraya koenigii*	−10.5	0.3281
4	IMPHY008900	Withaphysalin D	*Physalis minima*	−10.5	0.3088
5	IMPHY010666	Withanolide I	*Withania somnifera*	−10.5	0.3182
6	IMPHY000366	Jervine	*Veratrum viride*	−10.4	0.3355
7	IMPHY011452	Delavinone	*Fritillaria delavayi*	−10.4	0.3467
8	IMPHY009067	Picrasidine N	*Picrasma quassioides*	−10.4	0.2811
9	IMPHY002700	Withaphysalin A	*Physalis minima*	−10.3	0.3029
10	IMPHY005170	Withanolide O	*Withania somnifera*	−10.3	0.3121
11	BL-918			−9.4	0.2611

Ligand efficiency (LE) is reported in kcal/mol per non-hydrogen atom, representing the normalized docking score relative to ligand size. BL-918, is included as the known reference ULK1 activator for comparative assessment.

### 3.2 ADMET properties

The pharmacokinetic and toxicological properties of small molecules are key considerations in the drug discovery and development process (Pagan et al., 2019). ADMET is an analysis of potential therapeutics that helps estimate their drug-likeness, bioavailability, and safety (Ferreira and Andricopulo, 2019). The computational methods can also be successfully used to predict these properties, thereby saving time and cost in experimental studies. The pharmacokinetic parameters of the selected 10 compounds were further screened with Deep-PK (Myung et al., 2024). From the screened ten compounds, AMDET analysis identified four compounds, Withametelin B, Candidine, Jervine, and Delavinone, with promising pharmacokinetic profiles (Table 2). The compelling finding from the study was that all four phytoconstituents exhibit permeability across the blood-brain barrier (BBB), a key physicochemical property for the discovery of drug molecules targeting ALS.

**TABLE 2:** ADMET parameters of the selected compounds.

S. No.	Phytochemical and control molecule	Absorption	Distribution	Metabolism	Excretion	Toxicity	Carcinogenicity
		GI absorption	BBB permeation	CYP2C6 inhibitor	OCT2 substrate	AMES/Hepatotoxicity	
1	Withametelin B	High	Yes	No	No	No	No
2	Candidine	High	Yes	No	No	No	No
3	Jervine	High	Yes	No	No	No	No
4	Delavinone	High	Yes	No	No	No	No
5	BL-918	Low	No	Yes	No	No	No

Gastrointestinal (GI) absorption, blood-brain barrier (BBB) permeability, cytochrome P450 enzyme inhibition, renal clearance, toxicity predictions, and Carcinogenicity assessment.

### 3.3 PASS analysis

PASS analysis was performed to explore the potential biological activities of the screened hits, Withametelin B, Candidine, Jervine, and Delavinone. Evaluating the biological activities of small molecules is a crucial step in drug discovery, as it predicts whether compounds will exhibit the intended pharmacological attribute. The PASS analysis can be used to extract two important values: Pa, the likelihood that the compound is active for a particular function, and Pi, the possibility of inactivity (Lagunin et al., 2000). PASS analysis indicated that two of the four molecules screened, Candidine and Delavinone, have relevant biological activities. These compounds exhibited a range of Pa values of 0.944–0.143, indicating considerable biological potential relevance in ALS and kinase-associated pathways (Table 3).

**TABLE 3:** Biological activity predictions of the selected compounds based on the PASS (Prediction of Activity Spectra for Substances) analysis.

S. No.	Phytochemical	Structure	Pa	Pi	Activity
1	Withametelin B	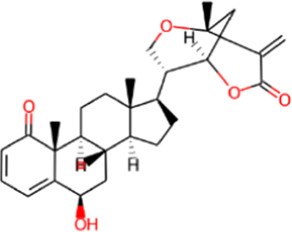	0.968	0.004	Antineoplastic
			0.524	0.018	Transcription factor stimulant
			0.310	0.056	Antibacterial
			0.307	0.073	Antipsoriatic
			0.263	0.032	Antioxidant
2	Candidine	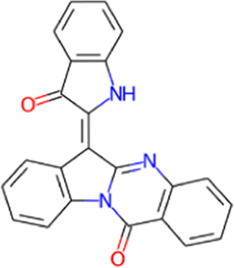	0.804	0.005	Kinase modulator
			0.512	0.105	Antineurotic
			0.512	0.105	Antineurotic
			0.262	0.149	Antiinflammatory
			0.253	0.119	Antineurogenic pain
3	Jervine	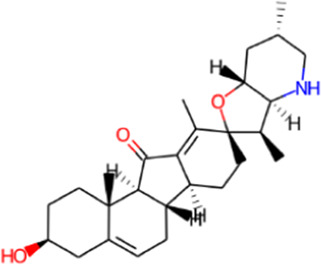	0.944	0.001	Hedgehog signaling inhibitor
			0.722	0.022	Antineoplastic
			0.503	0.022	Transcription factor stimulant
			0.385	0.065	Analeptic
			0.143	0.112	Anesthetic
4	Delavinone	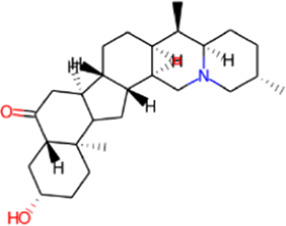	0.897	0.004	Respiratory analeptic
			0.799	0.005	Analeptic
			0.622	0.006	Antinociceptive
			0.563	0.018	Polarisation stimulant
			0.460	0.025	Dementia treatment
5	BL-918	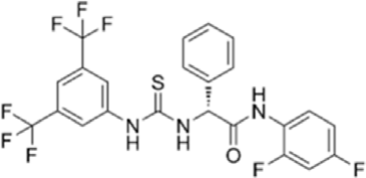	0.737	0.002	Glycine transporter inhibitor
			0.284	0.013	Antileprosy
			0.144	0.010	Autophagy inducer
			0.220	0.088	TRKB antagonist
			0.365	0.253	Nootropic

Pa: Probability to be active; Pi: Probability to be inactive. A higher Pa value indicates a more substantial likelihood of the compound exhibiting the associated biological effect.

### 3.4 Interaction analysis

**FIGURE 1:**
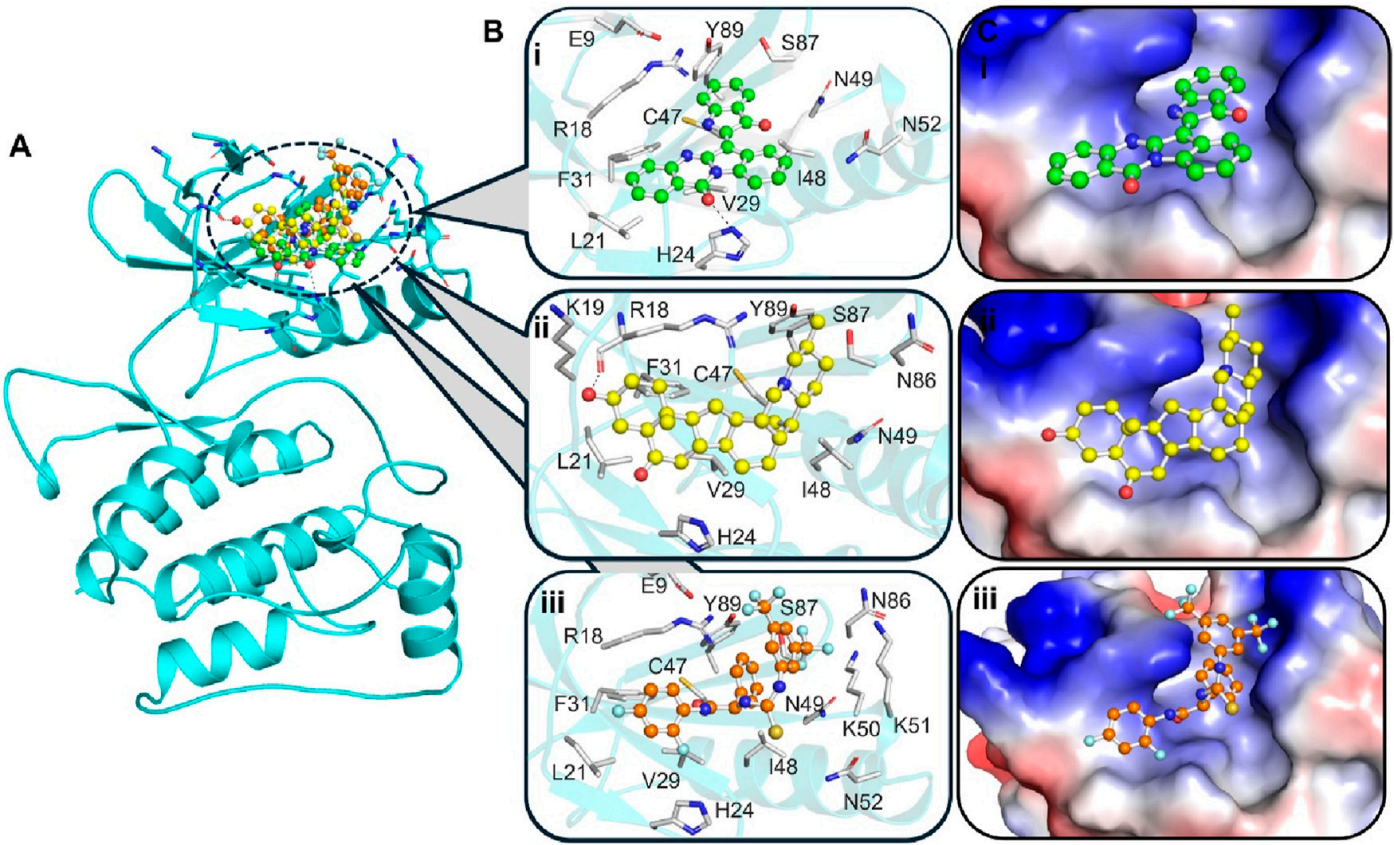
Molecular interactions between ULK1 and the ligands Candidine, Delavinone, and BL-918. The binding pocket of ULK1 is shown in cyan, with the ligands represented in distinct colors: Candidine (green), Delavinone (yellow), and BL-918 (orange). **(A)** Displays the binding poses of all ligands within the ULK1 binding site. **(B)** Provides a magnified view of key hydrogen bonding and hydrophobic interactions between ULK1 and the ligands. **(C)** Presents an electrostatic surface representation of ULK1, highlighting ligand binding and the spatial orientation of the ligand within the binding pocket.

Interaction analysis was conducted to investigate the binding mechanism of Candidine and Delavinone with ULK1. The docked confirmation files of both compounds were extracted and analyzed for their interaction with key residues in the ULK1 structure. Since the binding site is essential for the functional activity of ULK1, the binding behavior of Candidine and Delavinone at this site was of particular interest. The structural studies revealed strong binding complementarity between both compounds and the binding site pocket of ULK1, providing a favorable binding orientation (Figure 1). It was demonstrated that Candidine and Delavinone fit snugly within the binding site cavity, indicating potential activation of ULK1 (Figure 1A). A detailed investigation into molecular interactions revealed that both compounds exhibit persistent interactions with corresponding amino acid residues, including Arg18, Leu21, Val29, Cys47, Ile48, Ser86, and Tyr89 (Figure 1B). These interactions closely resembled those of known ULK1 activators, endowing these compounds with the potential to act as modulators of ULK1 (Ouyang et al., 2018). Structural analysis further revealed that Candidine and Delavinone bound inside the deep binding pocket of ULK1, suggesting that these compounds may act as activating ligands and mediate a modification in ULK1 that promotes its activation (Figure 1C). These results demonstrate the ability of Candidine and Delavinone as potential ULK1-targeting molecules, which merit further therapeutic evaluation.

**FIGURE 2:**
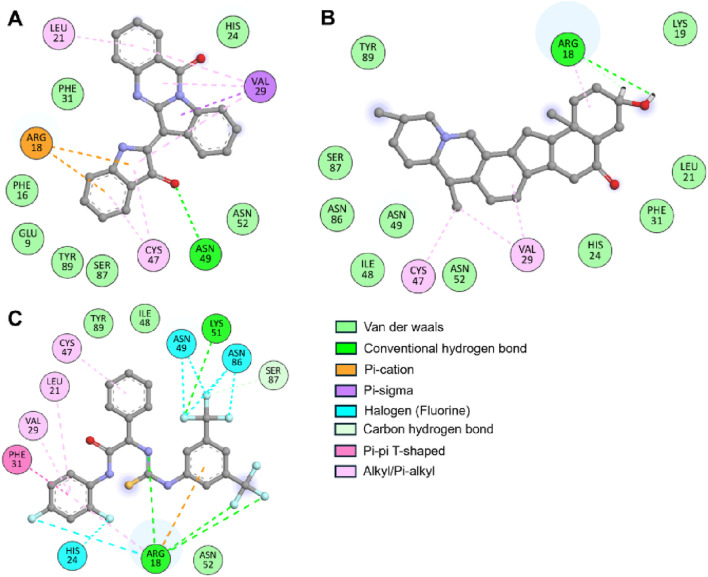
Two-dimensional (2D) interaction diagrams showing the molecular interactions of ULK1 with phytochemicals. **(A)** Candidine, **(B)** Delavinone, and **(C)** BL-918. Hydrogen bonds, hydrophobic interactions, and π-π stacking interactions are illustrated using standard color-coded symbols (green lines for hydrogen bonds, purple for π-π stacking, etc.). Ligand atoms are shown in ball-and-stick format, and interacting amino acid residues are labeled. These visualizations were generated using Discovery Studio Visualizer to depict binding orientation and key contact residues.

The crystal structure of ULK1 (PDB ID: 6QAS) includes a well-defined ATP-binding pocket and key residues such as Arg18, Leu21, Cys47, Ser86, and Tyr89 that contribute to ligand recognition. These residues were found to be critical in ligand interactions with Candidine and Delavinone, suggesting the importance of the kinase domain in stabilizing the ligand-protein complex. The binding modes of Candidine and Delavinone were compared with those of the reference ULK1 activator BL-918 using Discovery Studio Visualizer to perform a detailed interaction analysis. We generated 2D interaction diagrams to illustrate all potential molecular interactions, providing insight into the key residues involved in binding (Figure 2). The docking analysis revealed that both Candidine and Delavinone formed multiple interactions within the binding site of ULK1, thus exhibiting strong binding affinity towards the target region (Figures 2A,B). The compounds were found to have similar key binding interactions with BL-918, as observed in the plots, suggesting a similar mode of action (Figure 2C).

### 3.5 DFT analysis

**FIGURE 3:**
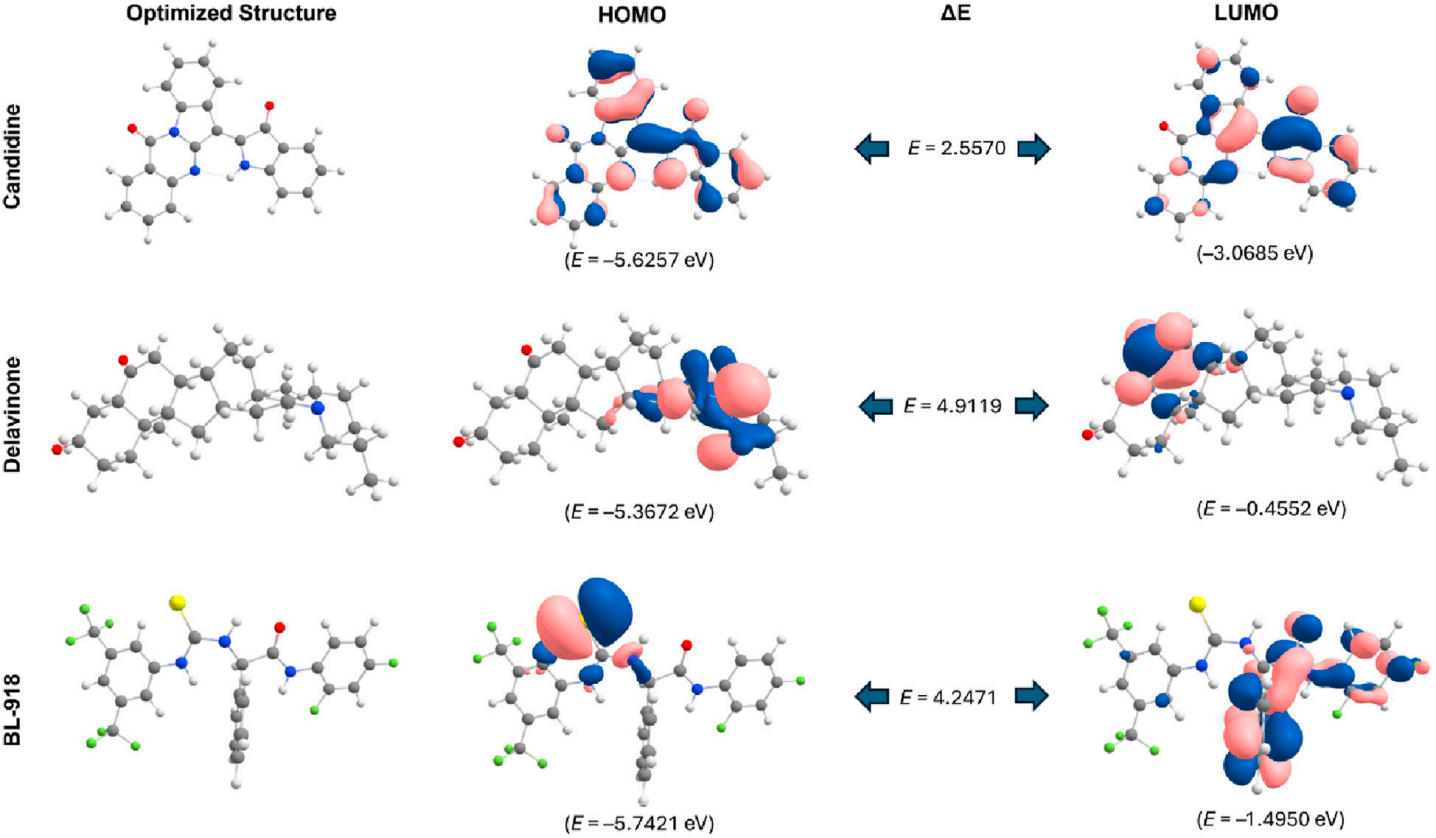
HOMO-LUMO representations and energy gaps of Candidine, Delavinone, and BL-918 derived from DFT analysis. The energy difference between the HOMO and LUMO is also illustrated in the figure, with values expressed in electronvolts (eV). Blue and pink represent the positive and negative phases of the molecular orbital wavefunction, respectively.

DFT is a powerful tool for developing novel organic corrosion modulators in modern drug discovery (Obot et al., 2015). We performed DFT calculations for the optimized geometries and electronic properties of Candidine, Delavinone, and BL-918. These evaluations were made based on the HOMO and LUMO, as a measure of the electronic reactivity of the compounds. HOMO is the highest energy orbital accessible to the system to donate an electron, while LUMO is the lowest energy receiving orbital to accept an electron in the process. The energy gap of HOMO and LUMO (Δ*E* H-L) gives information about the stability and reactivity behaviour of the compound. A narrow energy gap indicates a high reactivity, because a low energy is required for the transition of electrons, while a higher gap, less reactivity and more stability. It can be seen from Figure 3 that Candidine had higher reactivity with a HOMO-LUMO energy gap of 2.5570 eV. The Delavinone HOMO–LUMO gap was 4.9119 eV, while 4.2471 eV for the HOMO–LUMO gap of BL-918. The HOMO and LUMO distributions and their energy level are shown in Figure 3. The orbital maps show the electron profile, and blue and pink correspond to the positive and negative lobes of the molecular orbitals.

### 3.6 MD simulations analysis

MD simulations were performed to elucidate the thermodynamic properties and binding behavior of ULK1 in its free and complex forms with Candidine and Delavinone. The simulations were performed for 300 ns, providing detailed insights into the conformational stability and flexibility of ULK1 after its binding to the ligands. The stability of ULK1 in the presence of the Candidine and Delavinone was further examined using the various structural parameters described in the following subsections.

#### 3.6.1 Structural dynamics analysis

**FIGURE 4:**
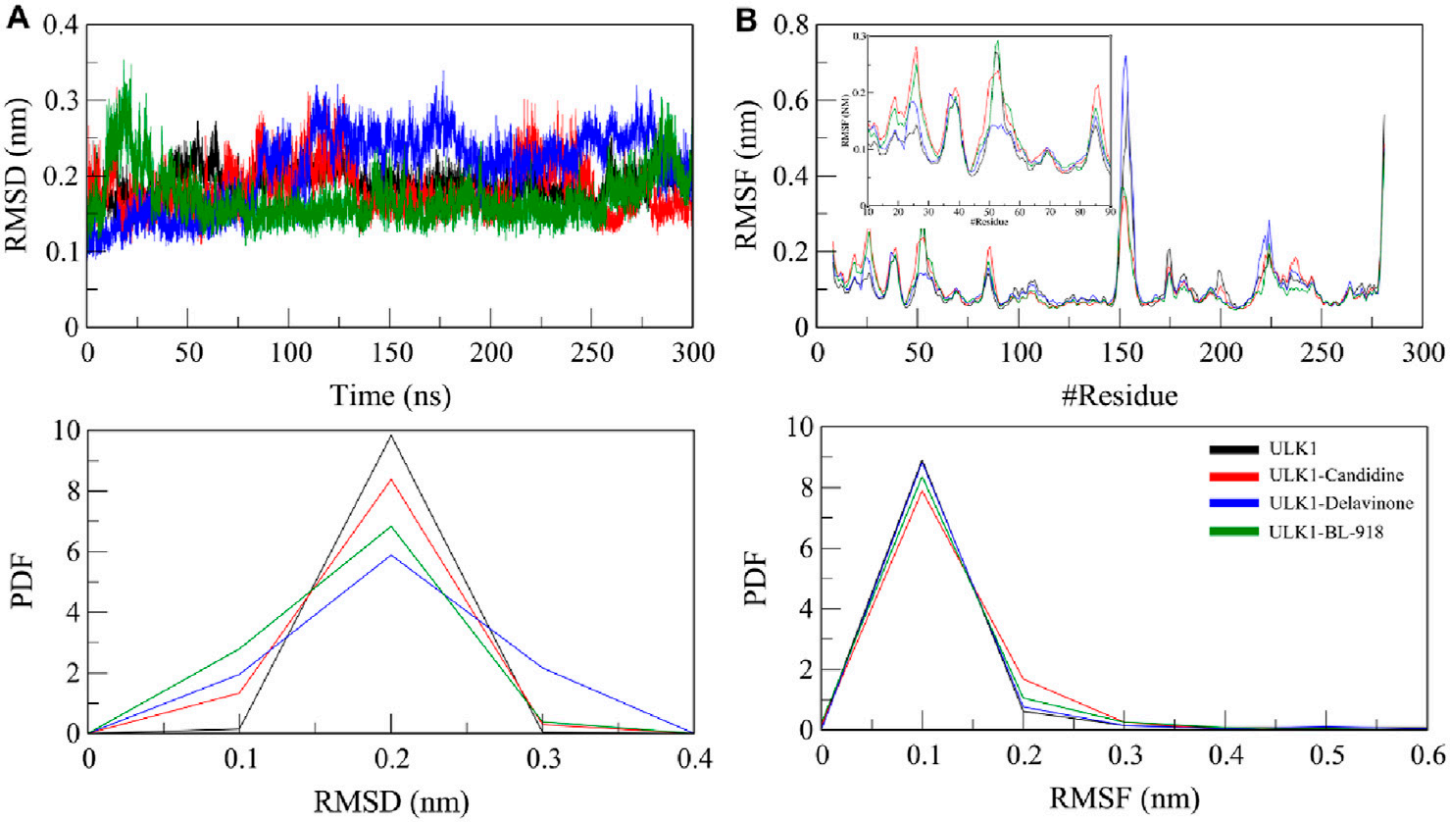
Structural dynamics of ULK1 in its free and ligand-bound states. **(A)** Root mean square deviation (RMSD) plots showing the stability of ULK1, ULK1-Candidine, ULK1-Delavinone, and ULK1-BL-918 complexes over the 300 ns simulation period. **(B)** Root mean square fluctuation (RMSF) plots depicting residual flexibility within the protein structure before and after ligand binding. Subgraph in panel B shows RMSF pattern of the ligand binding region in ULK1, i.e., amino acid residues 10-90. The lower panels present probability distribution function (PDF) values.

Root mean square deviation (RMSD) and root mean square fluctuation (RMSF) analyses were conducted to explore the structural dynamics of ULK1 upon complexing with the screened compounds (Figure 4). This RMSD metric is the standard for evaluating total protein stability in terms of atomic positional shifts throughout the simulation (Pikkemaat et al., 2002). The RMSD values for ULK1 in its free state, ULK1-Candidine, ULK1-Delavinone, and ULK1-BL-918 complexes were observed to be 0.19, 0.18, 0.21, and 0.17 nm, respectively. The results showed that the protein retained its structural stability throughout the 300 ns simulation, with only minor deviations (Figure 4A). While slight differences were observed in the trajectory of the RMSD of the ULK1-Delavinone complex during the middle of the simulation time, these variations were not significant. They indicated that ligand binding had not introduced any considerable instability in terms of conformation. Furthermore, the appearance of the RMSD probability distribution function (PDF) was assessed and confirmed a slight change for ULK1-Delavinone versus ULK1, suggesting a very slight but consistent expansion of protein dynamics upon ligand binding (Figure 4A, lower panel).

To examine the local flexibility of ULK1 further upon docking with Candidine and Delavinone, an RMSF analysis was performed on the simulated trajectories. RMSF quantifies the movement of each amino acid residue, enabling the identification of regions of flexibility and rigidity. The computed average RMSF values were found to be 0.11, 0.11, 0.11, and 0.10 nm for ULK1, ULK1-Candidine, ULK1-Delavinone, and ULK1-BL-918, respectively, which suggests that ULK1 exhibited consistent fluctuation behavior before and after binding with the ligand (Figure 4B). There were only minor changes for the RMSF values of ULK1-Candidine and ULK1-Delavinone, indicating local conformational adjustments to the added ligand, while the overall structural stability of the protein was preserved. Analysis of RMSF PDFs confirmed that these fluctuations were within reasonable ranges, lending further credibility to the stability of the ligand-bound complexes (Figure 3B, lower panel).

#### 3.6.2 Structural compactness analysis

**FIGURE 5:**
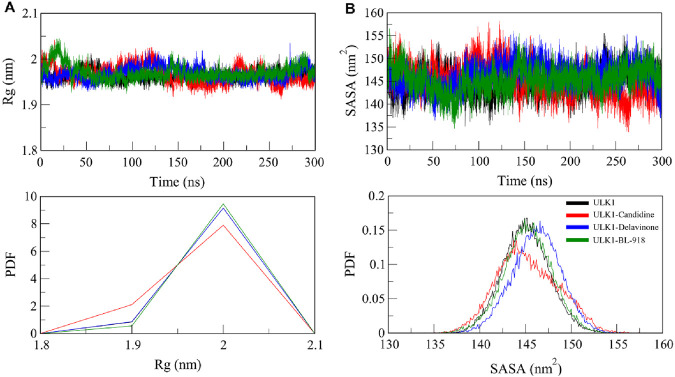
Compactness and folding stability of ULK1 upon ligand binding. **(A)** Radius of gyration (*R*_g_) plot, representing the overall compactness of ULK1 in its free and ligand-bound forms throughout the simulation. **(B)** Solvent-accessible surface area (SASA) plot, illustrating changes in protein solvent exposure due to ligand binding. Lower panels display the PDF values for *R*_g_ and SASA.

The radius of gyration (*R*_g_) is a widely used measure of the compactness of a protein that was calculated to determine the overall integrity of ULK1 upon ligand binding. *R*_g_ provides insights into a protein’s folding state by evaluating the distribution of atomic mass around its center of mass, with lower *R*_g_ values indicating a more compact structure (Lobanov et al., 2008). The *R*_g_ of ULK1, ULK1-Candidine, and ULK1-Delavinone complexes during the 300 ns long trajectory run of ULK1, ULK1-Candidine, ULK1-Delavinone, and ULK1-BL-918 complexes were found to be 1.96, 1.97, 1.97, and 1.97 nm, respectively. A minor increase in *R*_g_ for ULK1-Candidine and ULK1-Delavinone could reflect a slight structural expansion that can be predicted due to the occupancy of a few intramolecular spaces by the ligands, occupying portions within the protein (Figure 5A). This was also reflected in the PDF of *R*_g_ values, demonstrating that the overall structure was stable and indicated a well-folded ligand-bound state, confirming the stability of the ULK1 fold in the presence of Candidine and Delavinone (Figure 5A, lower panel).

Further, the solvent-accessible surface area (SASA) of ULK1 and its complexes was calculated and plotted to assess the stability of these structures under solvent conditions. SASA is a quantitative measure of a biomolecule’s exposure to the solvent in its surrounding environment, reflecting both conformational stability and molecular interactions (Durham et al., 2009). The average SASA values were calculated as 145.04, 145.25, 146.21, and 145.25 nm^2^ for ULK1, ULK1-Candidine, ULK1-Delavinone, and ULK1-BL-918, respectively (Table 4). We observed a small increase in SASA for ligand-bound complexes, indicating minor changes in the solvent accessibility of the protein upon ligand binding (Figure 5B). Nonetheless, the protein remained stable throughout the entire simulation, showing no significant change. The PDF distribution of SASA values subsequently demonstrated that the changes are within a permissible range, thereby further supporting the structural stability of ULK1-Candidine and ULK1-Delavinone complexes (Figure 5B, lower panel).

**TABLE 4:** Average molecular dynamics (MD) parameters for free ULK1 and its ligand-bound complexes.

Protein/protein-ligand system	RMSD (nm)	RMSF (nm)	*R*g (nm)	SASA (nm^2^)	#Intramolecular H-bonds
ULK1	0.19	0.11	1.96	145.04	196
ULK1-Candidine	0.18	0.11	1.97	145.25	200
ULK1-Delavinone	0.21	0.11	1.97	146.21	201
ULK1-BL-918	0.17	0.10	1.97	145.25	201

Parameters include root mean square deviation (RMSD, in nanometers), root mean square fluctuation (RMSF, in nanometers), radius of gyration (*R*g, in nanometers), solvent-accessible surface area (SASA, in nm^2^), and the average number of intramolecular hydrogen bonds over the 300 ns simulation. These metrics reflect the structural stability, compactness, and solvation properties of ULK1 in its apo and holo forms.

#### 3.6.3 Dynamics of hydrogen bonds

**FIGURE 6:**
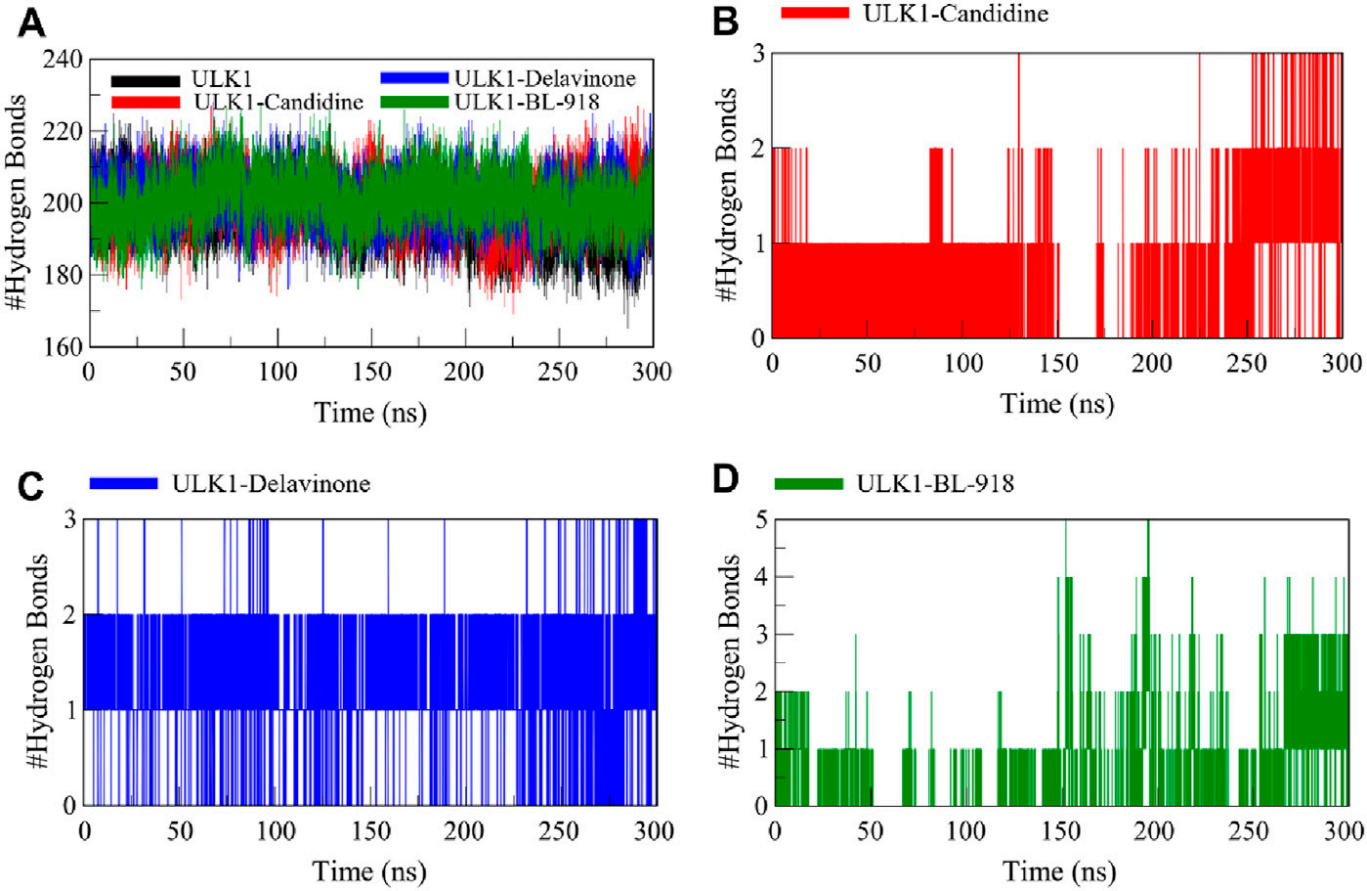
Intramolecular and intermolecular hydrogen bond formation between ULK1 and the ligands Candidine, Delavinone, and BL-918. **(A)** Time progression of intramolecular hydrogen bonds in ULK1, showing the stability of the protein’s internal hydrogen bonding network during the simulation. **(B)** Time progression of hydrogen bonds between ULK1 and Candidine, demonstrating the stability and frequency of ligand-protein interactions. **(C)** Time progression of hydrogen bonds between ULK1 and Delavinone. **(D)** Time progression of hydrogen bonds between ULK1 and BL-918.

Hydrogen bonding is fundamental to protein conformations and protein-ligand binding, and the characteristics of hydrogen bonding are highly informative of the stability and mode of interaction of a ligand with a protein (Williams and Ladbury, 2003). To analyze the effects of ligand binding on ULK1 conformational stability, the hydrogen bonds of ULK1 in free and ligand-bound form were examined from the simulation trajectories (Figure 6). Before and after the formation of the complex of ULK1 with Candidine and Delavinone, the number of hydrogen bonds formed in ULK1 was tracked and plotted to analyze for any significant differences (Figure 6A). The average number of hydrogen bonds was calculated as 196, 200, 201, and 201 for ULK1, ULK1-Candidine, ULK1-Delavinone, and ULK1-BL-918, respectively. The analysis also showed that the change in the number of intramolecular hydrogen bonds in ULK1 was negligible upon ligand binding. The complex formation did not show statistically significant perturbation with respect to the ULK1 structure.

Further analysis was conducted to assess the intermolecular hydrogen bonds formed by ULK1 with Candidine and Delavinone. The ULK1-Candidine interaction displayed 1 to 3 intermolecular hydrogen bonds, suggesting moderate binding stability during simulation (Figure 6B). Similarly, the ULK1-Delavinone complex also formed between 1 and 3 intermolecular hydrogen bonds during the simulation (Figure 6C). At the same time, the ULK1-BL-918 complex formed up to 5 intermolecular hydrogen bonds in a time-delayed way (Figure 6D). Overall, intermolecular hydrogen bond distribution showed that the ULK1-Delavinone complex exhibited a more stable hydrogen bond distribution than the ULK1-Candidine complex, indicating more substantial and persistent ligand-protein interactions.

#### 3.6.4 Secondary structure dynamics

**FIGURE 7:**
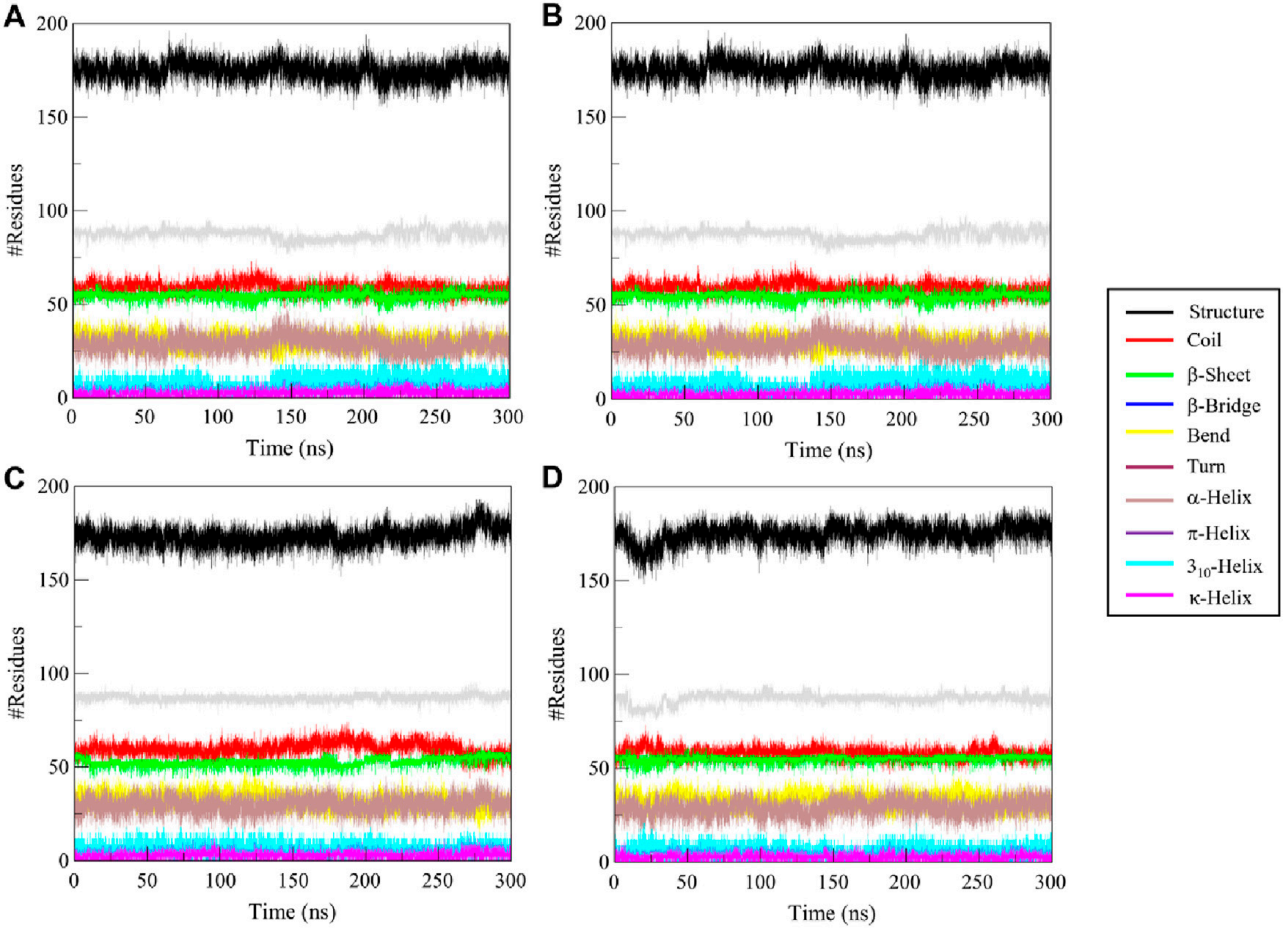
Time-resolved secondary structure elements for **(A)** free ULK1, **(B)** ULK1–Candidine, **(C)** ULK1–Delavinone, and **(D)** ULK1–BL-918 during the 300 ns simulation.

To study the structural effects of ligand binding to ULK1, we investigated the secondary structure elements along the 300 ns simulation. Changes in secondary structure elements (α-helix, β-sheet, turn, coil) may indicate flexibility or conformational change induced by ligand binding. Figure 7 shows the time-dependent secondary structure populations by free ULK1 and by its complexes with Candidine, Delavinone, and BL-918, and the average composition of each structure is given in Table 5. ULK1 showed a consistent secondary structure element distribution throughout the simulation (Figure 7A). A higher turn and helix content was found to be present in the ULK1–Candidine complex, suggesting a more flexible structure (Figure 7B). It was also accompanied by a small decrease in the amount of coil and bend formation. On the other hand, the content of α-helices residues of the ULK1-Delavinone complex became higher, and most structure did not change (Figure 7C). The secondary structure profile of the ULK1–BL-918 complex was almost unchanged (Figure 7D). Together, these minor structural variations suggest that ligand binding induces only mild conformational changes in ULK1, without causing significant destabilization or impairing its functional integrity.

**TABLE 5:** Average distribution of secondary structure elements in ULK1 and its ligand-bound complexes over a 300 ns simulation.

Secondary structure element	ULK1	ULK1-candidine	ULK1-delavinone	ULK1-BL-918
Coil	61	59	60	58
β-sheet	55	55	52	55
β-bridge	3	3	3	4
Bend	33	30	32	33
Turn	27	29	30	29
α-helix	86	87	87	87
π-helix	0	0	0	0
3_10_-helix	6	7	6	5
κ -helix	3	2	2	3

The number of residues adopting each secondary structure type, coil, β-sheet, β-bridge, bend, turn, α-helix, 310-helix, and π-helix, was averaged across all trajectory frames. These data provide insights into the conformational stability and flexibility of ULK1 upon ligand binding.

### 3.7 Principal component analysis

**FIGURE 8:**
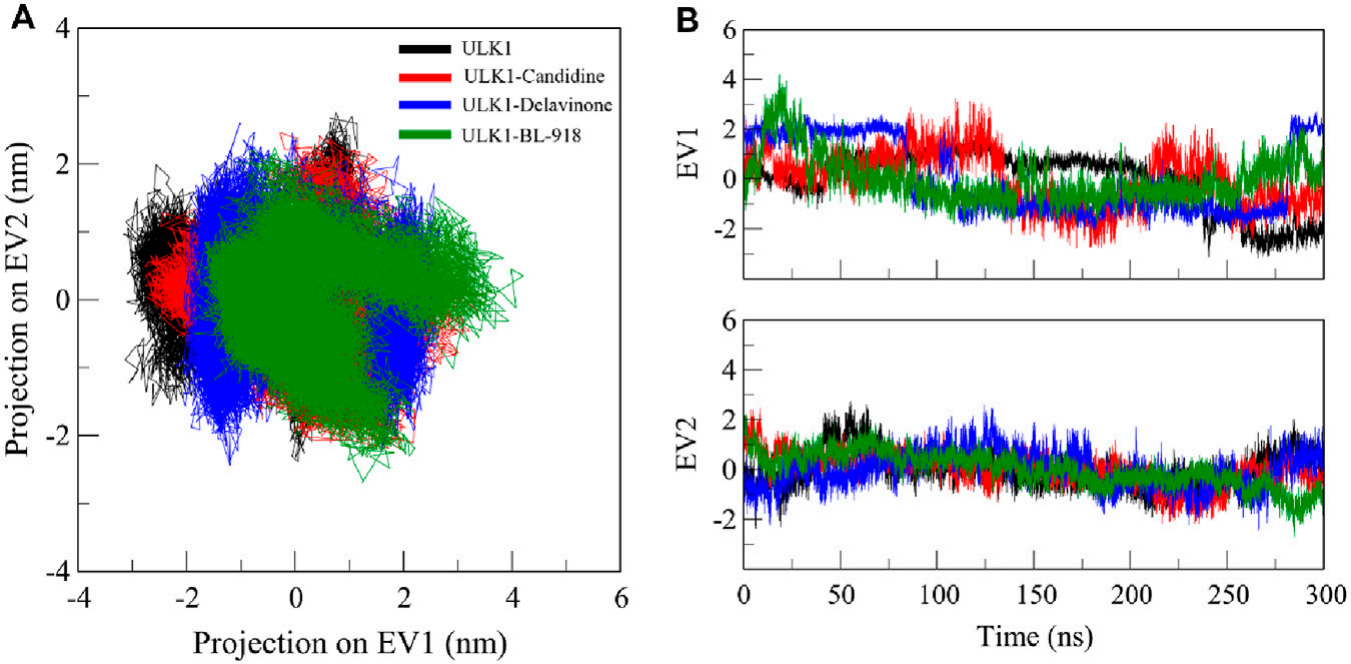
Principal component analysis (PCA). **(A)** 2D projections illustrating the essential dynamics of ULK1, ULK1-Candidine, ULK1-Delavinoneand, and ULK1-BL-918. **(B)** Time-dynamics projection highlighting the dominant conformational changes observed in each system.

PCA is a widely used method for essential dynamics analysis, which identifies the conformational movements of proteins and protein-ligand systems, reducing high-dimensional data into principal components that capture the dominant motion patterns of the biomolecule (Yang et al., 2009). We performed PCA to analyze the collective motions of the ULK1 protein and its complexes with Candidine and Delavinone during simulations (Figure 8). The PCA analysis revealed that the ULK1 protein and its ligand-bound forms exhibited distinct projections along two principal eigenvectors (EVs) calculated from the Cα atomic displacements (Figure 8A). The enrichment analysis revealed that ULK1-Delavinone exhibited a more compact projection than ULK1, ULK1-Candidine, and ULK1-BL-918. The ligand-bound complexes showed stable conformational motions, with minor deviations from the conformation of the native state, even with diverse normative trajectories (Figure 8B). The PCA results showed clustering of the ligand-bound complexes compared to free ULK1, indicating ligand-induced restriction of conformational mobility. Such clustering reflects stable and restricted motions, consistent with binding-induced stabilization.

### 3.8 Free energy landscape analysis

**FIGURE 9:**
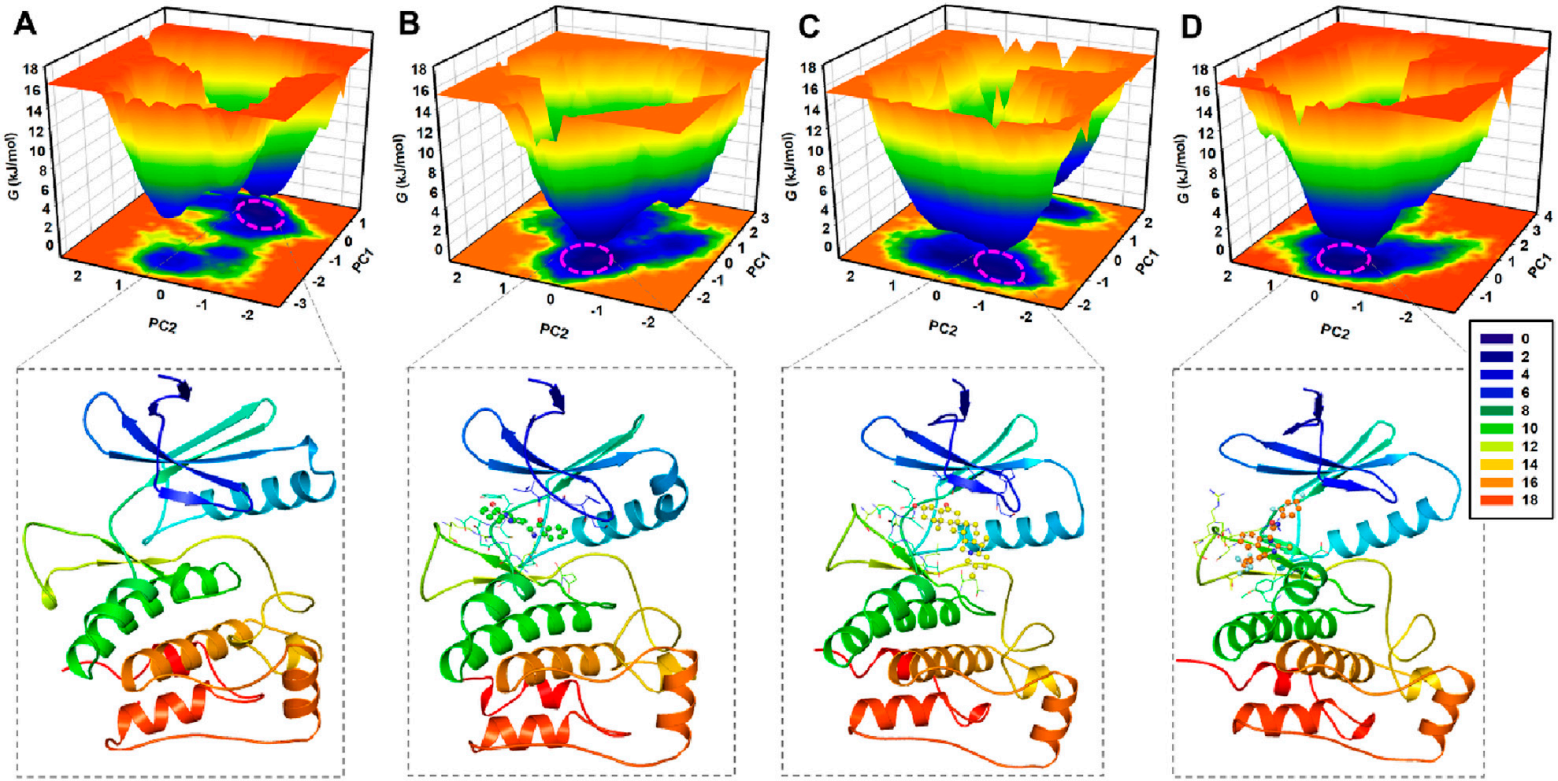
Free energy landscape (FEL) plots of ULK1 in different states. **(A)** FEL of ULK1, displaying a single global minimum corresponding to the most stable protein conformation. **(B)** FEL of the ULK1-Candidine complex, showing shifts in energy basins due to ligand binding. **(C)** FEL of the ULK1-Delavinone complex with multiple stable conformational states. **(D)** FEL of the ULK1-BL-918 complex, revealing multiple stable conformational states. The deeper blue regions represent the most energetically favorable conformations, providing insights into the thermodynamic stability of ULK1-ligand interactions. The lower panels showed the fetched protein structural snapshot from the global minima.

FEL analysis is another valuable technique for evaluating the impact of ligand binding on protein folding and conformational stability (Abdelsattar et al., 2021). FEL provides insights into the folding dynamics by mapping the energy states of a protein through simulations, revealing the most stable regions and the extent of reshaping in response to ligand binding. To assess the interaction of Candidine and Delavinone with elongating ULK1, FELs were generated from the PCA trajectories to visualize the global minima and folding mechanism of ULK1. Energy links to ligand-bound and unbound conformations of ULK1 that are presented in deep blue on the contour plots represent the states that are energetically preferred and nearest to the respective native state (Figure 9). ULK1 presented a single global minimum, stable in 1-2 basins (Figure 9A). In complexes with Candidine and Delavinone, the expansion of energy basins was observed in the FELs, where ULK1 adopted diverse stable conformations with two to three global minima (Figures 9B,C). At the same time, ULK1-BL-918 also adopted diverse stable conformations with a single global minimum (Figure 9D). The FEL structural analysis revealed distinct conformational preferences of ULK1 in its unbound and ligand-bound states (Figure 9, lower panels). Structural snapshots extracted from the global minima highlight the most stable conformations, offering mechanistic insights into how each ligand modulates ULK1 stability and flexibility.

### 3.9 MM-PBSA analysis

To evaluate the binding affinities of the studied ligands for ULK1, we carried out MM/PBSA calculations for the protein-ligand complexes using the production trajectories. These studies dissected the binding energy (Δ*G*_binding_) into contributions from the gas-phase (van der Waals (vdW) and electrostatic) and solvation (polar and non-polar) effects. The results show that Candidine and Delavinone exhibited better binding energy as compared to BL-918 (Table 6). Concretely, ULK1-Delavinone reached the lowest binding free energy (19.99 ± 3.73 kJ/mol), with the next being ULK1-BL-918 (−18.26 ± 2.71 kJ/mol) and ULK1-Candidine (−6.93 ± 2.79 kJ/mol). The negative values indicate a strong interaction and stability of ULK1 with the selected phytochemicals during the binding process. Overall, the data signify the possibility of Candidine and Delavinone as potential ULK1 binders are worth deeper validation.

**TABLE 6:** MM/PBSA-derived binding energy components for ULK1-ligand complexes.

Complex	*ΔG*_VDWAALS_	*ΔE*_EL_	*ΔE*_PB_	*ΔE*_NPOLAR_	*ΔG*_GAS_	*ΔG*_SOLV_	${∆G}_{Total}\left(kJ/mol\right)$
ULK1-Candidine	−15.03	−3.82	13.69	−1.77	−18.85	11.92	−6.93 ± 2.79
ULK1-Delavinone	−40.66	−13.19	38.40	−4.54	−53.85	33.86	−19.99 ± 3.73
ULK1-BL-918	−35.71	0.89	20.38	−3.82	−34.82	16.56	−18.26 ± 2.71

## 4 Discussion

Targeting ULK1 is an emerging strategy for mitigating the pathological accumulation of toxic protein aggregates characteristic of ALS (Ouyang et al., 2018). Given the limitations associated with existing synthetic ULK1 activators, including BL-918, our focus on bioactive phytoconstituents aims to provide safer, structurally novel, and more druggable candidates. This study identifies and characterizes two phytochemicals, Candidine and Delavinone, as promising natural activators of ULK1 using an integrated computational approach. The docking analysis suggests that the screened phytoconstituents have high binding potential towards ULK1 and may be further investigated as potential modulators of ULK1 activity in ALS. The top-ranked compounds represented structurally diverse scaffolds, reflecting chemical classes such as isoquinoline alkaloids (e.g., Candidine) and steroidal alkaloids (e.g., Delavinone). This diversity increases the chance of identifying novel binding modes and provides broader applicability for therapeutic development. ADMET findings indicate that Withametelin B, Candidine, Jervine, and Delavinone possess drug-like properties and non-toxic features and should proceed to the next stage of the screening cascade as ULK1 activators relevant to ALS therapeutics. The PASS analysis showed that both Candidine and Delavinone exhibited biological activities, including kinase-modulating, antineurotic, anti-inflammatory, and antineurogenic pain-relieving properties, as well as potential treatments for dementia symptoms. This enabled the identification of promising candidates for further validation and optimization.

The interaction analysis Key residues, such as Arg18 and Cys47, are directly involved in the binding with Candidine and Delavinone, suggesting that these compounds may be promising modulators of ULK1. Considering that they directly interact with the same residues used by BL-918, Candidine and Delavinone may enhance ULK1 activity, as found in previous findings (Ouyang et al., 2018; Liu et al., 2023). The interaction analysis provides insight into the molecular mechanisms of these compounds and further establishes their potential as future drug candidates after further validation. The MD simulation results suggest that the binding of Candidine and Delavinone to ULK1 is not accompanied by significant structural perturbations or instability. These analysis methods, specifically RMSD and RMSF, contribute essential information that helps determine the atomic-level dynamics of ULK1, reiterating the roles of Candidine and Delavinone as promising hit modulators of ULK1 for future drug development.

The *R*_g_ and SASA findings imply that binding of Candidine and Delavinone induces minimal conformational rearrangement, resulting in negligible compromise of structural compactness. The consistent patterns observed in both *R*_g_ and SASA analyses underscore the dynamic stability of these ligand-bound states, highlighting Candidine and Delavinone as stable modulators of ULK1 activity. The intramolecular hydrogen bond analysis of ULK1 and ULK1-ligand complexes showed a higher number of hydrogen bonds, confirming that the internal hydrogen bond network of ULK1 was preserved and more compact. While the intermolecular hydrogen bond dynamics showed Candidine and Delavinone form stable hydrogen bond interactions with ULK1, further stabilizing the complex. The ULK1-Delavinone complex, on the other hand, showed more stability than Candidine and ULK1. These findings of intermolecular interactions provide valuable information for the development and optimization of novel ULK1 modulators for therapeutic potential.

PCA analyses confirmed that the global motion of ULK1 was minimally affected by ligand binding, suggesting that the structure of both complexes remained stable within proximity throughout the simulation for Candidine and Delavinone. These results also offer key insights into the dynamics of ULK1 and its interaction with the screened compounds, paving the way for future studies on their translational utility. FEL analysis suggests that the binding of ligands induces slight conformational changes without altering the native folding landscape of the protein. No indication of unfolding was observed, indicating that ULK1 remained stable during the simulation. The FEL results showed that Candidine and Delavinone only affected a slight conformational state of ULK1, but no significant destabilization was observed. A limitation of this study is the reliance on a single best-docked pose for each ligand as the starting conformation in MD simulations. Although commonly practiced, this approach may not capture the full conformational flexibility.

Overall, these findings support the use of Candidine and Delavinone as structurally novel and pharmacokinetically viable activators of ULK1. Notably, Delavinone showed superior MM-PBSA-derived binding affinity (−19.99 ± 3.73 kJ/mol), stable hydrogen bonding, and minimal structural perturbation of ULK1, positioning it as a particularly compelling lead candidate. This study is limited by its reliance on computational data without experimental validation. The data support their advancement toward preclinical validation. Further studies should investigate their autophagy-inducing activity and neuroprotective effects in relevant ALS cellular and animal models, as well as structure-activity relationship (SAR) analysis for lead optimization. In summary, these results suggest that both ligands interact with ULK1 without compromising the structural integrity of the target, indicating them as promising candidates for further assessment as ULK1 modulators.

## 5 Conclusion

ALS is a progressive neurodegenerative disease characterized by the accumulation of toxic protein aggregates, including mutant SOD1, which leads to the degeneration of motor neurons. Since the clearance of these aggregates is mediated by autophagy, improving autophagic pathways has been considered a possible therapeutic approach. ULK1 is a key activator of autophagy and is a promising target for drug development in ALS. This study was designed to identify bioactive phytoconstituents that activate ULK1 using a computational drug discovery approach. Multi-step virtual screening was conducted against a library of phytochemicals sourced from the IMPPAT 2.0 database. Structure-based molecular docking revealed that Candidine and Delavinone have the most potent binding affinities for the ULK1 binding site, compared to BL-918, a known ULK1 activator. Analysis of ADMET showed that both compounds exhibited good ADMET properties, including BBB permeability, without any observed toxicity. Their potential biological activity was further supported by PASS analysis, which revealed various relevant activities. DFT and MD simulations were conducted to assess the stability of ULK1 in a complex with Candidine and Delavinone. The RMSD, RMSF, *R*_g_, and SASA analysis results indicated that binding the ligand caused only minor structural changes while the overall stability of the protein was preserved. Hydrogen bond interactions exhibited greater stability in the complex, particularly with ULK1 and Delavinone. PCA, as well as FEL and MM-PBSA analyses, also confirmed that ULK1’s folding mechanism was not substantially impacted by ligand binding. These small-molecule compounds, Candidine and Delavinone, possess strong binding potential towards ULK1 and advantageous drug-like properties. These structurally diverse phytochemicals present promising candidates for further experimental validation in autophagy-related ALS therapy development. The results offer valuable insights into the therapeutic potential of ULK1 modulation by phytochemicals and call for subsequent *in vitro* and *in vivo* evaluations for drug development.

## Data Availability

All data supporting the findings of this study are available within the paper. The phytochemicals and protein structures analyzed are publicly available from IMPPAT (https://cb.imsc.res.in/imppat/) and the RCSB Protein Data Bank (https://www.rcsb.org/structure/6QAS).
